# Carbon Dots-Modified Nanoporous Membrane and Fe_3_O_4_@Au Magnet Nanocomposites-Based FRET Assay for Ultrasensitive Histamine Detection

**DOI:** 10.3390/molecules24173039

**Published:** 2019-08-22

**Authors:** Yijie Mao, Yu Zhang, Wei Hu, Weiwei Ye

**Affiliations:** 1 Institute of Ocean Research, Zhejiang University of Technology, Hangzhou 310014, China; 2 Department of Food Science and Technology, Zhejiang University of Technology, Hangzhou 310014, China

**Keywords:** fluorescence resonance energy transfer, CDs, nanoporous alumina membrane, food safety

## Abstract

Histamine can be formed by enzymatic decarbonylation of histidine, which is an important indicator of seafood quality. A rapid and sensitive assay method is necessary for histamine monitoring. A fluorescence resonance energy transfer (FRET) assay system based on a carbon dot (CD)-modified nanoporous alumina membrane and Fe_3_O_4_@Au magnet nanocomposites has been developed for histamine detection in mackerel fish. CDs immobilized on nanoporous alumina membranes were used as donors, which provided a fluorescence sensing substrate for histamine detection. Fe_3_O_4_@Au magnet nanocomposites can not only act as acceptors, but also concentrate histamine from fish samples to increase detection sensitivity. Histamine was detected by the fluorescence signal changes of CDs capturing histamine by an immune reaction. The fluorescence signals of CDs were quenched by Fe_3_O_4_@Au magnet nanocomposites via the FRET mechanism. With an increase of histamine, the fluorescence intensity decreased. By recording fluorescence spectra and calculating intensity change, histamine concentration can be determined with a limit of detection (LOD) of 70 pM. This assay system can be successfully applied for histamine determination in mackerel fish to monitor the fish spoilage process in different storage conditions. It shows the potential applications of CDs-modified nanoporous alumina membranes and Fe_3_O_4_@Au magnet nanocomposites-based biosensors in the food safety area.

## 1. Introduction

Histamine is naturally synthesized in the human body and is important for physiological functions. It regulates neurotransmission and hormone secretion, and affects food intake and cardiovascular control [1,2,3]. Histamine can be formed by enzymatic decarbonylation of histidine in some fish, such as sardines, tuna and mackerel fish, that are improperly stored or transported [4]. Once histamine is formed, it cannot be destroyed to any great extent by conventional cooking and freezing methods. Histamine enters the human body through the food chain. An excess level of histamine in the blood is related to pathological conditions such as mastocytosis, gastric acid disorders, and neuropsychiatric disorders [5]. Histamine is frequently regarded as one of the food quality indicators during the processes of producing, storing, transporting and transacting food, and it is therefore essential to monitor trace levels of histamine continuously.

Conventional histamine detection relies on chromatographic analysis, high-performance liquid chromatography (HPLC), capillary zone electrophoresis and enzyme-linked immunosorbent assay (ELISA) [6,7,8]. These detection methods can obtain highly accurate results, but they need expensive instrumentation and sophisticated techniques, and require qualified technicians. Miniaturized and integrated biosensors, such as nanofluidic sensors, cell-based assays, and quartz crystal microbalance (QCM)-based sensors, have been studied for rapid detection of histamine in food [9,10]. They provide sensitive and rapid histamine detection platforms with low cost but they are limited by electrode properties. Fluorescence resonance energy transfer (FRET) biosensors provide a promising and useful tool for small molecule detection based on the energy transfer between donors and acceptors [11]. 

Fluorescent dyes and proteins are often used as the donor and acceptor pairs. They have the disadvantages of photobleaching and poor chemical stability. The emergence of photo-stable fluorescence nanoparticles, such as semiconductor quantum dots (QDs) and upconversion nanoparticles (UCNPs), overcomes these shortcomings, but their high toxicity limits their applications in biological areas. Fluorescent carbon dots (CDs) are chemically stable and have low toxicity [12,13,14]. Together with easy surface functionalization and high brightness, CDs have attracted increasing attention as donors in FRET biosensors [15,16,17]. 

Nanoporous membranes have nano-ordered structures, high surface-to-volume ratio, and the properties of easy fabrication and surface modification. They have been used to detect proteins, bacteria, virus, cancer cells, and food toxins [18,19]. Due to the standard fabrication and non-conductive frame with nanopores, nanoporous alumina membranes are appropriate candidates for establishing FRET biosensors for histamine determination. 

The good magnetic manipulability of magnetic nanoparticles (MNPs) makes them unique in separation and analysis [20]. Gold nanoparticles (AuNPs) are easy to synthesize with well-controlled diameters and surface modification. They are chemically stable and have special optical properties [21]. AuNP and MNP nanocomposites conjugated by chemical bonding can be used for protein separation and bacterial detection [22]. Fe_3_O_4_@Au magnet nanocomposites have attracted much research interest because they integrate the properties and advantages of both MNPs and AuNPs. Fe_3_O_4_@Au magnet nanocomposites have unique surface plasmon resonance (SPR) and magnetic manipulability. They are widely applied in biosensing and immunoassays in nanomedicine [23,24,25].

In this study, we report on a sensitive system based on a CD-modified nanoporous alumina membrane and Fe_3_O_4_@Au magnet nanocomposites for histamine determination in mackerel fish samples. The system is based on the FRET assay with CDs as donors and Fe_3_O_4_@Au magnet nanocomposites as acceptors. We found that Fe_3_O_4_@Au magnet nanocomposites concentrated histamine from the samples, which was captured by the CDs, triggering FRET phenomena due to the close distance between the CDs and Fe_3_O_4_@Au magnet nanocomposites. The fluorescence intensity of CDs was quenched by Fe_3_O_4_@Au magnet nanocomposites. By calibrating the histamine concentration using this FRET system, the histamine concentration was determined in mackerel fish samples. The proposed FRET system can detect histamine in the range of 0.1 nM to 1 mM with a limit of detection (LOD) of 70 pM. It can be used for histamine detection in mackerel fish in different storage conditions and time slots, showing the potential for food safety investigation.

## 2. Results

### 2.1. Mechanism of Histamine Detection by the FRET System

**Figure 1:**
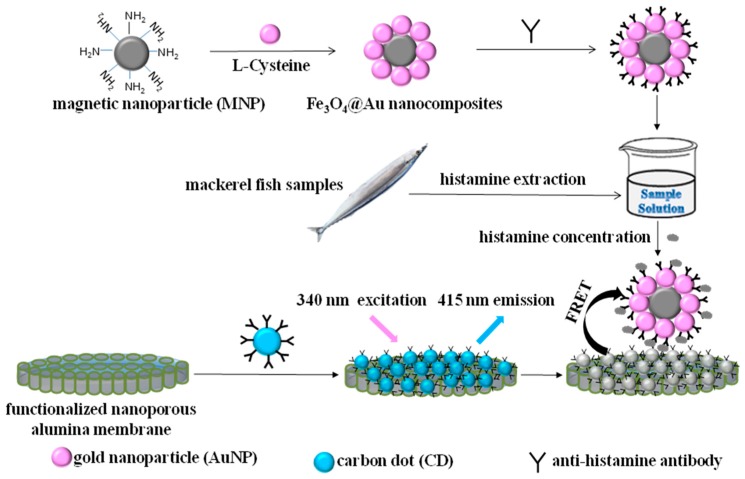
Schematic diagram of histamine extraction and concentration using the CD-modified nanoporous alumina membrane and Fe_3_O_4_@Au magnet nanocomposite-based FRET system for histamine determination.

The mechanism of the FRET system based on CD-modified nanoporous membrane/Fe_3_O_4_@Au magnet nanocomposites for histamine detection is shown in Figure 1. Nanoporous alumina membranes were functionalized by (3-glycidyloxypropyl) trimethoxysilane (GPMS) and immobilized with CDs with anti-histamine antibody modification. CDs were the donors of the FRET system. MNPs were conjugated with AuNPs by l-cysteine to form Fe_3_O_4_@Au magnet nanocomposites, which acted as the acceptors with magnetic properties. Fe_3_O_4_@Au magnet nanocomposites were functionalized by anti-histamine antibody and concentrated histamine from the sample solution. The Fe_3_O_4_@Au magnet nanocomposites with histamine were separated from the samples by a magnetic field. The capture of histamine by anti-histamine antibody brought Fe_3_O_4_@Au magnet nanocomposites close to the CDs. Under 340 nm excitation, the emission spectrum of CDs is shown in Figure S1a. The maximum emission is at 450 nm. The absorption spectrum of Fe_3_O_4_@Au magnet nanocomposites presented a wide absorption range between 450 nm and 700 nm (Figure S1b). When Fe_3_O_4_@Au magnet nanocomposites were brought close to CDs, the emission of the CDs was transferred to the Fe_3_O_4_@Au magnet nanocomposites and quenched, leading to a FRET process on the membrane. The fluorescence intensity decreased.

### 2.2. Characterization of Magnetic Nanoparticles and Nanoporous Alumina Membranes

**Figure 2:**
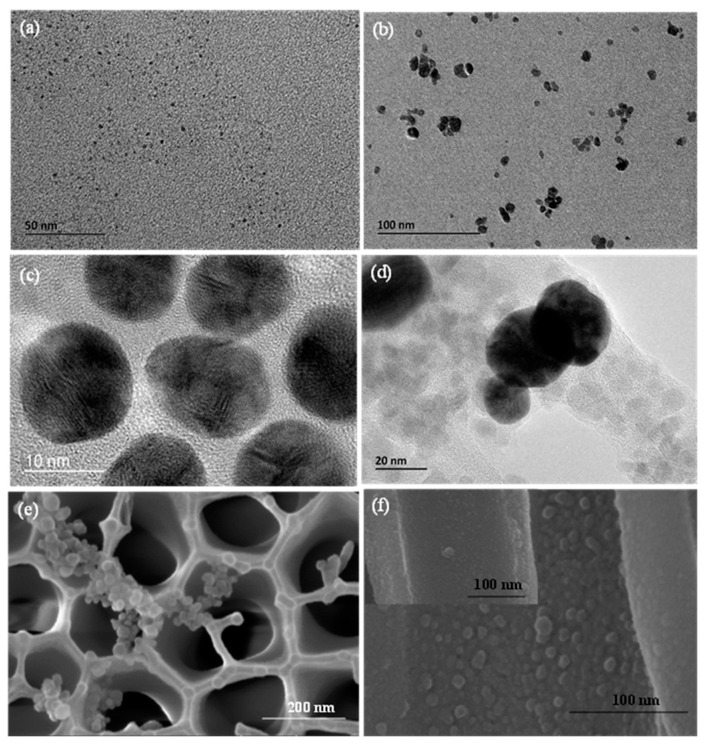
(**a**) TEM image of CDs. (**b**) TEM image of MNPs. (**c**) TEM image of AuNPs. (**d**) TEM image of Fe_3_O_4_@Au magnet nanocomposites. (**e**) SEM image of Fe_3_O_4_@Au magnet nanocomposites concentrating histamine to the CD-modified nanoporous alumina membrane. (**f**) Cross-sectional view of the nanoporous alumina membrane with histamine concentrated by Fe_3_O_4_@Au nanocomposites and without histamine (inset).

TEM was used to characterize the morphology and sizes of the CDs, MNPs, AuNPs and Fe_3_O_4_@Au magnet nanocomposites (Figure 2). Figure 2a shows good monodispersity of CDs in DI water. The average diameter ranges from 2 nm to 5 nm. The MNPs and AuNPs dispersed well in DI water with an average diameter of 20 nm (Figure 2b) and 10 nm (Figure 2c), respectively. AuNP was connected to the edge of MNP to form the Fe_3_O_4_@Au magnet nanocomposite (Figure 2d). Anti-histamine antibody-modified Fe_3_O_4_@Au magnet nanocomposites were used to conjugate histamine and concentrate histamine from the fish samples. They were captured by the antibody-modified CDs, which were immobilized on nanoporous membranes. SEM images showed the surface morphology of nanoporous alumina membranes with histamine captured by the nanoparticles on the nanoporous membrane (Figure 2e). The bare nanoporous alumina membrane had a honeycomb-like structure. The surface became rough with clusters of nanoparticles aggregated on the membrane due to the capture of histamine. Figure 2f (inset) indicates that several small CD particles formed a thin layer in the nanopores. When the Fe_3_O_4_@Au magnet nanocomposites concentrated histamine and added to the substrate, multilayers were observed because of histamine capture by the antibody on the CDs (Figure 2f). As a result, the CDs and the Fe_3_O_4_@Au magnet nanocomposites were observed in the walls of the nanopores. The close distance of the Fe_3_O_4_@Au magnet nanocomposites and CDs could lead to the FRET effect on the nanoporous alumina membrane surface and in the nanopores.

**Figure 3:**
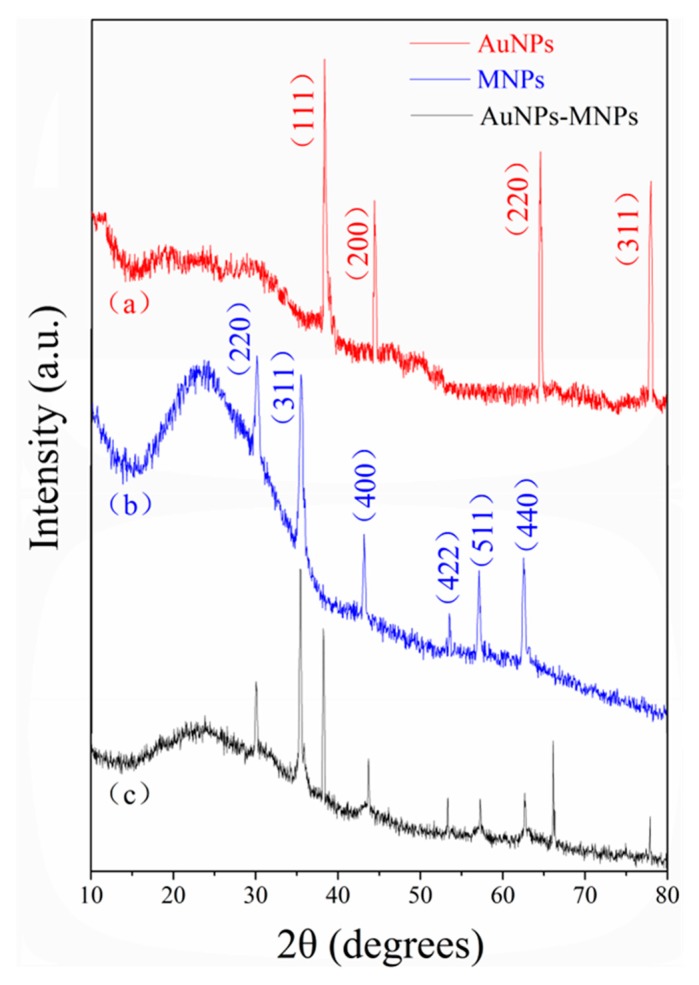
XRD patterns for (**a**) AuNPs, (**b**) MNPs, and (**c**) Fe_3_O_4_@Au nanocomposites.

The XRD patterns of AuNPs, MNPs and Fe_3_O_4_@Au nanocomposites are shown in Figure 3. The XRD spectra of the synthesized AuNPs (Figure 3a) matched the pattern of standard gold nanoparticles well with reference to the crystal structures from the JCPDS No. 04-0784 [26]. Four typical peaks at 38.3°, 44.4°, 64.6° and 78.0° corresponded to the AuNP crystal planes (111), (200), (220) and (311), respectively. The MNP diffraction pattern (Figure 3b) displayed planes (220), (311), (400), (422), (511) and (440) at 2θ values of 30.1°, 35.5°, 43.2°, 53.5°, 57.1° and 62.6°, respectively. The MNP peaks can be indexed to the face-centered cubic crystal structure of magnetite nanoparticles according to JCPDS No. 89-0691 [27]. In addition, all diffraction peaks attributable to AuNPs and MNPs were observed in the XRD spectra of the as-prepared nanocomposites (Figure 3c), indicating successful formation of Fe_3_O_4_@Au magnet nanocomposites.

The FTIR spectra were utilized to confirm the structure of Fe_3_O_4_@Au nanocomposites conjugated with histamine. As shown in Figure S2, the peak at 586 cm^−1^ is related to the characteristic absorption of the Fe−O bond from MNPs [28,29]. The peaks at 1635 cm^−1^ and 1113 cm^−1^ are attributed to the stretching vibration of the carbonyl group (C=O) and amide groups due to the reaction of carboxyl groups on the surface of MNPs with amino groups on l-cysteine, which introduces sulfhydryl groups from l-cysteine to MNPs for adsorption of AuNPs [30]. The presence of histamine can be demonstrated by the C=C stretching mode and C−H out-of-plane bending mode, which appeared at 1456.9 cm^−1^ and 875 cm^−1^, respectively [31,32]. The peaks at 2853 cm^−1^ and 2923 cm^−1^ are from the stretching vibration of −CH_2_− and −CH_3_. The FTIR spectra results demonstrated the successful conjugation of histamine by Fe_3_O_4_@Au nanocomposites.

### 2.3. Histamine Determination

**Figure 4:**
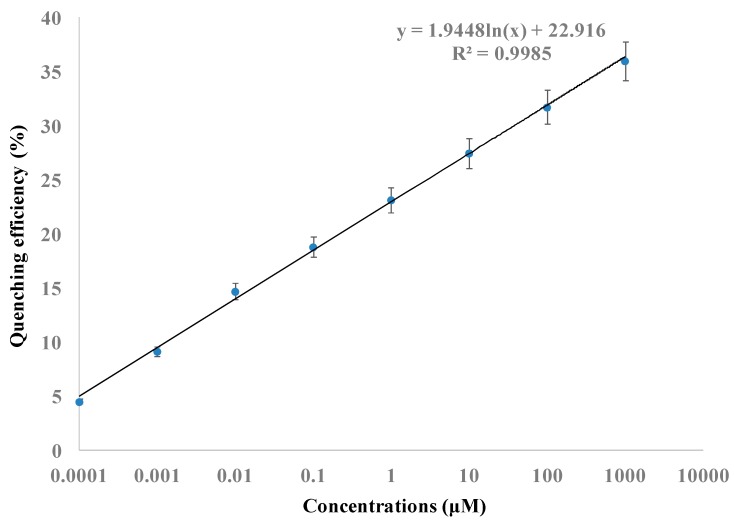
Linear relationship between quenching efficiency versus logarithmic concentrations of histamine.

Histamine was determined by the CDs-modified nanoporous membrane and Fe_3_O_4_@Au magnet nanocomposites-based FRET system. Antibody-modified Fe_3_O_4_@Au magnet nanocomposites were incubated with different concentrations of histamine for about 30 min to ensure conjugation between Fe_3_O_4_@Au magnet nanocomposites and histamine. The mixture was added to the antibody-modified CDs, which were immobilized on nanoporous alumina membranes. Histamine was captured by the antibody on the CDs. The fluorescence intensity was measured by a spectrophotometer. The fluorescence intensity of CDs without histamine was used as the control and the intensity was the largest because there was no quenching in the system. Fluorescence intensity decreased with the increase of histamine concentrations because more Fe_3_O_4_@Au magnet nanocomposites were brought close to CDs, leading to fluorescence quenching. Quenching efficiency was calculated by (F_0_ − F_q_)/F_q_ × 100%, where F_0_ was the fluorescence intensity of control detection and F_q_ was the fluorescence intensity of histamine detection. With the increase of histamine concentrations from 0.1 nM to 1 mM, the fluorescence intensity decreased and the quenching efficiency increased. As shown in Figure 4, a linear curve between fluorescence quenching efficiency and logarithmic histamine concentrations was obtained. The relationship equation is y = 1.9448ln(x) + 22.916, where y is the quenching efficiency and x is the histamine concentration. The limit of detection (LOD) was calculated based on the control signals plus three times the standard derivation, and it was 70 pM for histamine detection. Rotten food at different spoilage stages contains histamine ranging from tens of micromolars to hundreds of micromolars, and the United States Food and Drug Administration (FDA) established an advisory level of histamine lower than 50 ppm (around 450 µM) in fish for consumption [3,33]. The CDs-modified nanoporous membrane and Fe_3_O_4_@Au magnet nanocomposites-based FRET assay system showed promising potential in sensitive histamine detection in fish and seafood products to support food safety.

**Figure 5:**
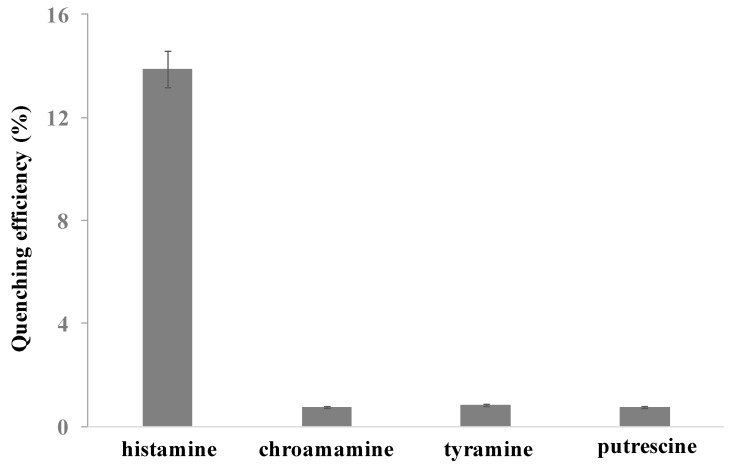
Comparison of the quenching efficiency of histamine, chroamamine, tyramine and putrescine detection.

To evaluate the specificity of this CDs-modified nanoporous membrane and Fe_3_O_4_@Au magnet nanocomposites-based FRET system for histamine detection, chroamamine, tyramine, and putrescine with the same concentration as histamine (10 nM) were measured with the same procedure. As shown in Figure 5, the quenching efficiency of histamine was 13.9%, while the quenching efficiency of chroamamine, tyramine, and putrescine was 0.7%, 0.8% and 0.8%, respectively. The quenching efficiency of chroamamine, tyramine, and putrescine was obviously lower than that of histamine. It demonstrated a good specificity of the CDs-modified nanoporous alumina membrane and Fe_3_O_4_@Au magnetic nanocomposites-based FRET assay system for histamine detection.

### 2.4. Histamine Detection in Mackerel Fish

**Figure 6:**
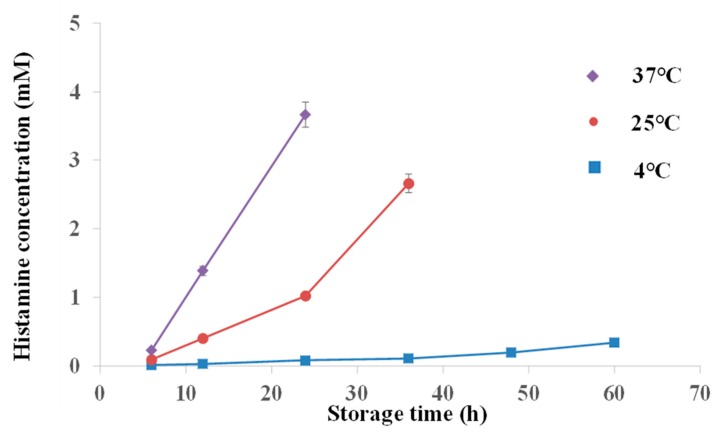
Histamine concentration determination with mackerel fish stored at 4 ℃, 25 ℃ and 37 ℃ over time.

The ability of the system for determining histamine concentrations in fish was evaluated. Histamine concentrations of fresh mackerel fish samples and mackerel fish samples that were stored at 4 ℃, 25 ℃ and 37 ℃ for 6 h to 60 h were tested. Fluorescence intensity decreased as the storage time increased for 4 ℃, 25 ℃ and 37 ℃ storage temperatures. The long storage time made fish rotten and produced histamine. The relationship between histamine concentrations and storage time is shown in Figure 6. Histamine concentrations increased with the increase of storage time under the three storage temperatures. When the mackerel fish was stored in a 4 ℃ environment for 6 h, the histamine concentration was 0.01 mM. It increased slowly to 0.3 mM in 60 h. The histamine concentration was 0.09 mM in mackerel fish stored in 25 ℃ for 6 h, and it quickly increased to 0.4 mM in 12 h. The histamine concentration of mackerel fish was 0.2 mM when they were stored at 37 ℃ for 6 h, and increased to 1.4 mM when they were stored at 37 ℃ for 12 h. The histamine concentrations were confirmed using the UV-Vis spectrometry method, which followed the Chinese national approved standard procedure (GB/T 5009.45-2003). The rapid increase in histamine in mackerel fish was due to quick deterioration in a high temperature environment. Compared to reported methods for histamine detection, such as cell-based assays, amperometric immunosensors, and surface plasmon resonance sensors, the FRET assay system based on CDs and Fe_3_O_4_@Au magnet nanocomposites presents the advantages of low LOD, good sensitivity and specificity [10,34,35].

## 3. Materials and Methods 

### 3.1. Materials 

Histamine (≥97%), sodium citrate tribasic dihydrate (≥99%), tryptamine (98%), tyramine (≥98.5%), 1,4-diaminobutane (99%), gold (III) chloride trihydrate (HAuCl_4_•3H_2_O, ≥99%), phosphate buffered saline (PBS, pH 7.4), 1-(3-dimethylaminopropyl)-3-ethylcarbodiimide hydrochloride (EDC) and GPMS (≥98%) were all bought from Sigma-Aldrich (St. Louis, MO., USA). Trichloroacetic acid (99%), 1-pentanol (98%), sodium hydroxide (97%), l-cysteine (99%) and glutaraldehyde (50%) were obtained from Aladdin Industrial Corporation (Shanghai, China). Nanoporous alumina membranes with a diameter of 13 mm were obtained from Whatman, Inc. (Maidstone, UK). The thickness of the membranes was 60 μm and the diameter of the nanopores was 200 nm. Carbon dots (CDs) were obtained from Beijing Beida Jubang Science and Technology Co. Ltd. (Beijing, China). Meso-2,3-dimercaptosuccinic acid (DMSA)-coated Fe_3_O_4_ nanoparticles (MNPs) were provided by Nanjing XFNANO Materials Tech Co. Ltd. (Nanjing, China). Monoclonal anti-histamine antibody produced in rabbit was supplied by Sigma-Aldrich. Ethanol (≥99.7%), hydrochloric acid (36–38%), nitric acid (65–68%), toluene (≥99.5%), hydrogen peroxide (≥30%) and acetone (≥99.5%) were all bought from Sinopharm Chemical Reagent Co. Ltd. (Shanghai, China). 

### 3.2. CDs Conjugated on Nanoporous Alumina Membranes

The surface modification procedures of nanoporous alumina membranes were conducted according to the previous study [36,37]. The membranes were immersed in boiling hydrogen peroxide (30%) for 30 min and rinsed in deionized (DI) water to clean the membranes and form reactive hydroxyl groups. The preprocessed membranes were dried and put in the mixed solution of toluene and GPMS (2%) at 60 °C overnight. The silane molecules of GPMS were conjugated onto the membranes. They were alternatively washed by toluene and anhydrous ethanol twice and followed by curing at 60 °C for 2 h.

To conjugate CDs on silanized nanoporous alumina membranes, glutaraldehyde, as a cross-link agent, was added into the CD solution and kept for 30 min at 4 °C. Monoclonal histamine antibody was then pipetted into the mixture for coupling to CDs at 4 °C. Then, the solution of CDs and antibody conjugation (30 μL) was added onto the silanized membranes at 4 °C overnight and anchored on the membranes by a chemical reaction between amino groups and epoxy groups. The final assemblies were stored at 4 °C for later use.

### 3.3. Fe_3_O_4_@Au Magnetic Nanocomposites Preparation and Functionalization

AuNPs can be synthesized according to the citrate reduction method with an average size of 10 nm. Freshly prepared aqua regia (mixture of HCl and HNO_3_ at a ratio of 3:1) was used to clean the beaker and stir bar, which were then rinsed by DI water before the experiment. HAuCl_4_ (12 μL, 14.3 wt%) was immediately dropped into the DI water (50 mL) that was boiling on a stirring hot plate with a magnetic stir bar. Sodium citrate solution (1%, 5 mL) was quickly put into the above mixture. The heating was not turned off until the solution color turned wine red. The solution was cooled down naturally while still stirring [38].

MNPs were dispersed in DI water (0.4 mg/mL, 60 μL). EDC was added to activate the MNPs. The solution was then kept for 30 min at 4 °C. MNPs were collected by magnetic separating, and subsequently re-dispersed in DI water. l-cysteine (6 μL, 1 M) was dropped into the above solution and kept at 4 °C overnight. l-cysteine can be bound to the surface of MNPs by the reaction between the carboxyl groups of MNPs and the amino groups of l-cysteine [39]. The sulfhydryl groups of l-cysteine were introduced to MNPs for adsorption of AuNPs. A mixture of AuNPs (600 μL) and l-cysteine-modified MNPs (60 μL) was kept at 4 °C overnight to form Fe_3_O_4_@Au nanocomposites. The nanocomposites were separated by a magnetic separator. The nanocomposites were rinsed in DI water. Antibody (6.6 μL, 2.1 mg/mL) was added to Fe_3_O_4_@Au magnet nanocomposites (600 μL) at 4 °C for about 10 h. Then, the antibody biofunctionalized nanocomposites were kept in PBS for later use.

### 3.4. Characterization

A transmission electron microscope (TEM, Jeol JEM-2100F, Tokyo, Japan), operating at 200 kV, was used to characterize the morphology and size of the CDs, MNPs, AuNPs and Fe_3_O_4_@Au nanocomposites. Samples for TEM were dropped on holey carbon-coated 400 mesh copper grids. A zeta potential analyzer (Brookhaven Omni, Austin, TX., USA) was used to measure the zeta potential. The morphology of nanoporous alumina membranes was observed by a scanning electron microscope (SEM, Hitachi SU8010, Tokyo, Japan), and the dry samples were coated with gold for 50 s. X-ray diffraction (XRD) patterns were collected on a PANalytical X’Pert PRO powder diffractometer (Almelo, The Netherlands) operated at 40 kV and 40 mA using Cu–Kα radiation. FTIR spectra were obtained with a Thermo Nicolet Nexus 670 spectrometer (Madison, WI. USA) employing KBr optics in the 4000–400 cm^−1^ region with a resolution of 2.0 cm^−1^.

### 3.5. Histamine Extraction from Mackerel Fish

Fresh mackerel fish kept in ice were purchased from the local fish market. The fish samples were wrapped in aseptic bags with ice. Mackerels were cleaned with the giblets removed. The samples were obtained from the homogenized fish weighing 10 g and mixed thoroughly with trichloroacetic acid (20 mL, 10%). The mixture was ultrasonic treated for 30 min and filtered using filter paper. The obtained filtrate (2 mL) was transferred to a funnel and adjusted to alkaline with sodium hydroxide. Histamine was extracted using 1-pentanol (3 mL) mixed with the filtrate for 5 min. The pentanol-extractable histamine was collected by separation. The extraction processes were performed three times. The extracts were collected and the volume was adjusted to 10 mL with 1-pentanol. Then the pentanol-extractable histamine (2 mL) was treated with hydrochloric acid (3 mL, 3%). The hydrochloric acid-extractable histamine was collected by separation. The extraction procedures were repeated three times and the extracts were gathered. The volume was adjusted to 10 mL with hydrochloric acid (3%) [40]. The pH of the final extracts was adjusted to 7.4 by sodium hydroxide.

### 3.6. Histamine Concentration and Detection

To detect histamine in mackerel fish stored under different conditions using the developed CDs-modified nanoporous membrane and Fe_3_O_4_@Au magnet nanocomposites-based assay system, standard histamine was detected for calibration. Histamine was diluted in PBS buffer to various concentrations from 10 pM to 500 mM. The suspension of anti-histamine antibody-modified Fe_3_O_4_@Au magnet nanocomposites (30 μL) were incubated with histamine at different concentrations for 30 min to ensure conjugation. They were concentrated by a magnetic field and added to the CDs-modified nanoporous alumina membranes and left for 2 h. Fe_3_O_4_@Au magnet nanocomposites conjugated with histamine were captured on nanoporous membranes. The membranes were rinsed by PBS to remove the unlinked magnetic nanocomposites. The fluorescence spectra of CDs on nanoporous alumina membranes were measured by a Hitachi F-2700 spectrophotometer (Tokyo, Japan) with 340 nm excitation to obtain the relationship between the fluorescence intensity value and the concentrations of histamine. The control experiments were performed by recording the fluorescence intensity of samples with addition of the same amount of PBS. To evaluate the selectivity of this CDs-modified nanoporous alumina membrane and Fe_3_O_4_@Au magnet nanocomposites-based system for histamine detection, 1,4-diaminobutane, tyramine and tryptamine were used for detection by the biosensor with the same procedure.

The samples of fresh mackerel fish were packed in sterile polyethylene bags (300 g/bag) and stored at 4 °C, 25 °C and 37 °C, respectively. The formation of histamine stored for 6 h to 60 h was monitored according to the above procedures. Histamine was extracted from fresh and stored mackerel fish and detected by the FRET assay system. The histamine concentrations were calculated from the relationship between the fluorescence intensity and histamine concentrations, which were calibrated by standard histamine. 

## 4. Conclusions

In this study, a novel FRET assay system based on CDs-modified nanoporous alumina membrane and Fe_3_O_4_@Au magnet nanocomposites was developed for histamine concentration and determination in fish. The FRET assay system was established by anti-histamine antibody-modified CDs immobilized on nanoporous alumina membranes. Fe_3_O_4_@Au magnet nanocomposites were prepared for histamine concentration and acted as the acceptors. This system can be successfully used for histamine concentration and detection ranging from 0.1 nM to 1 mM with a LOD of 70 pM. This FRET biosensor can be applied in histamine determination in fish samples and monitoring of the fish spoilage process in different storage conditions, showing promise as a tool for histamine detection to ensure food safety.

## Supplementary Materials

The following are available online at https://www.mdpi.com/1420-3049/24/17/3039/s1, Figure S1: (**a**) Emission spectrum of carbon dots (CDs); (**b**) absorption spectrum of Fe_3_O_4_@Au magnet nanocomposites. Figure S2: FTIR spectrum of Fe_3_O_4_@Au magnet nanocomposites conjugated with histamine.
