# Supplementary material for: Carbon Dots-Modified Nanoporous Membrane and Fe3O4@Au Magnet Nanocomposites-Based FRET Assay for Ultrasensitive Histamine Detection
Source: Molecules. 2019 Aug 22;24(17):3039. doi: 10.3390/molecules24173039 (PMC6749273; doi:10.3390/molecules24173039)
Supplement: Supplementary file 1 [file molecules-24-03039-s001.pdf]

Supporting Information

Carbon Dots-Modified Nanoporous Membrane and Fe_3O_4@Au Magnet Nanocomposites-Based FRET Assay for Ultrasensitive Histamine Detection

Yijie Mao [1,2], Yu Zhang [1,2], Wei Hu [1,2] and Weiwei Ye [1,2,*]

1 Institute of Ocean Research, Zhejiang University of Technology, Hangzhou 310014, China
2 Department of Food Science and Technology, Zhejiang University of Technology, Hangzhou 310014, China
* Correspondence: yeweiwei@zjut.edu.cn; Tel.: +86-0571-88332832

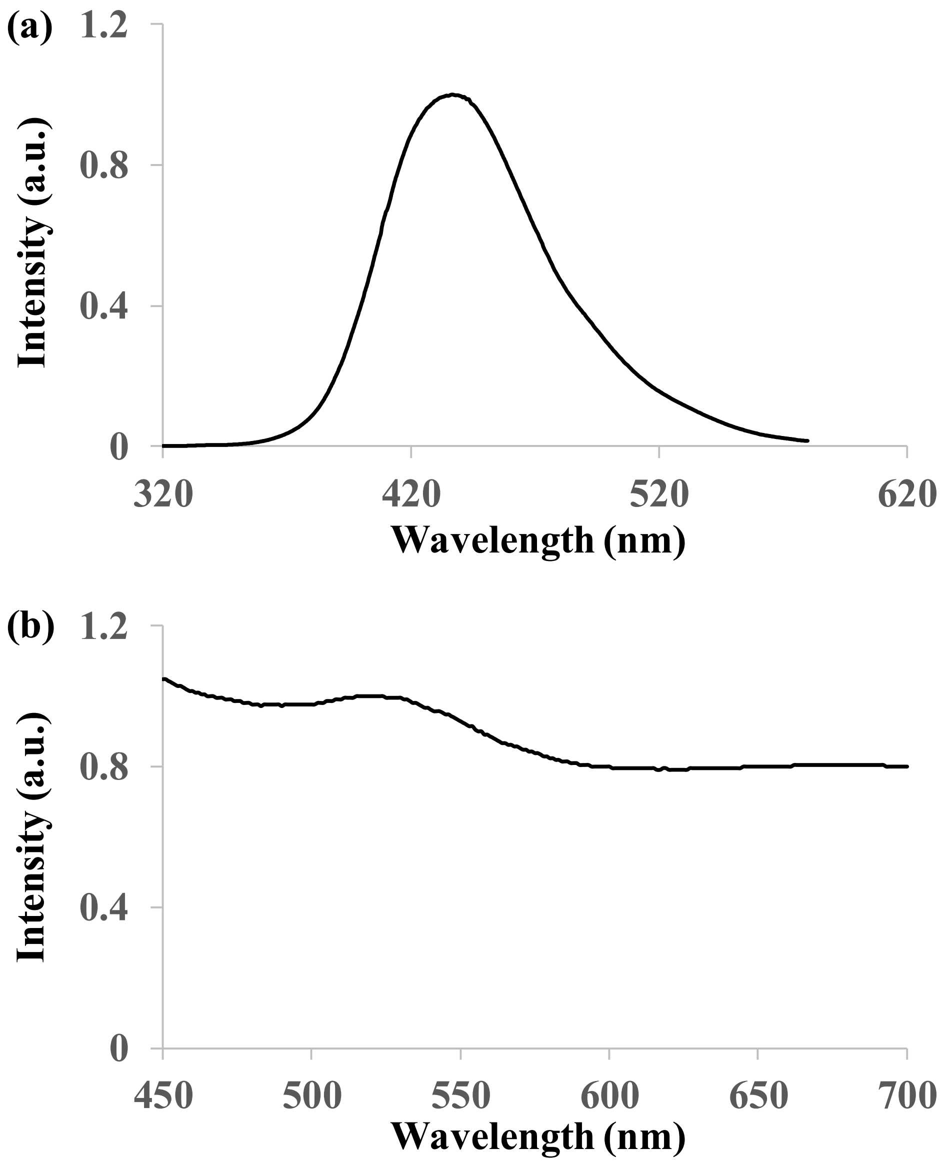

Figure S1. (**a**) Emission spectrum of carbon dots (CDs); (**b**) absorption spectrum of Fe_3O_4@Au magnet nanocomposites.

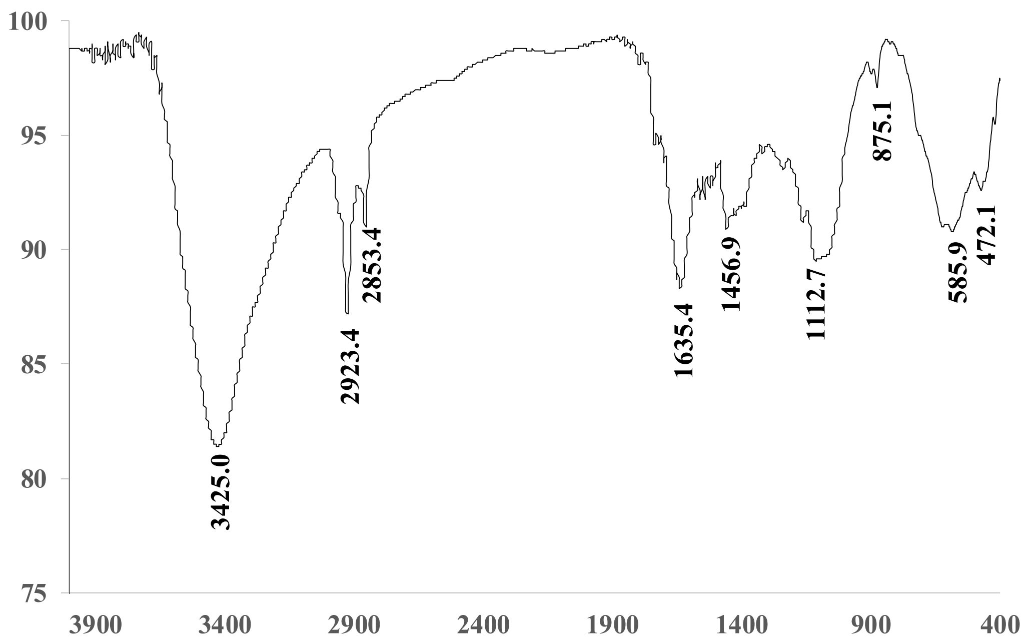

Figure S2. FTIR spectrum of Fe_3O_4@Au magnet nanocomposites conjugated with histamine.
